# S-Sulfocysteine Induces Seizure-Like Behaviors in Zebrafish

**DOI:** 10.3389/fphar.2019.00122

**Published:** 2019-04-02

**Authors:** Jennifer Plate, Wiebke A. Sassen, Ahmed H. Hassan, Franziska Lehne, Reinhard W. Köster, Tobias Kruse

**Affiliations:** ^1^Institute of Plant Biology, TU Braunschweig, Braunschweig, Germany; ^2^ Zoology Institute, Cellular and Molecular Neurobiology, TU Braunschweig, Braunschweig, Germany

**Keywords:** molybdenum cofactor, S-Sulfocysteine, sulfite oxidase, zebrafish, NMDA receptors

## Abstract

Sulfite is a neurotoxin, which is detoxified by the molybdenum cofactor (Moco)-dependent enzyme sulfite oxidase (SOX). In humans, SOX deficiency causes the formation of the glutamate analog S-Sulfocysteine (SSC) resulting in a constant overstimulation of ionotropic glutamatergic receptors. Overstimulation leads to seizures, severe brain damage, and early childhood death. SOX deficiency may be caused either by a mutated *sox* gene or by mutations in one of the genes of the multi-step Moco biosynthesis pathway. While patients affected in the first step of Moco biosynthesis can be treated by a substitution therapy, no therapy is available for patients affected either in the second or third step of Moco biosynthesis or with isolated SOX deficiency. In the present study, we used a combination of behavior analysis and vital dye staining to show that SSC induces increased swimming, seizure-like movements, and increased cell death in the central nervous system of zebrafish larvae. Seizure-like movements were fully revertible upon removal of SSC or could be alleviated by a glutamatergic receptor antagonist. We conclude that in zebrafish SSC can chemically induce phenotypic characteristics comparable to the disease condition of human patients lacking SOX activity.

## Introduction

Molybdenum (Mo) is an essential element for life (Mendel, 2013), which for biological activity requires being complexed within the molybdenum cofactor (Moco). Eukaryotic Moco synthesis is a multi-step pathway that ultimately combines the pterin part of the cofactor with molybdate (Mendel and Kruse, 2012). Moco is found in the active site of all eukaryotic molybdenum-dependent enzymes (Mo-enzymes) and hence defects in any of the genes involved in Moco biosynthesis result in the pleiotropic loss of all Mo-enzyme activities (Mendel, 2013). The corresponding human disease is termed as Moco deficiency [MoCD; (Johnson and Duran, 2001)]. The Mo-enzyme sulfite oxidase (SOX) is vitally important for humans (Kisker et al., 1997; Johnson and Duran, 2001; Schwahn et al., 2015). Without SOX activity, the degradation of sulfur (S)-containing amino acids is impaired (Kisker et al., 1997), and toxic sulfite is not detoxified. Sulfite reacts with cystin, thus forming S-Sulfocysteine (SSC). SSC is structurally highly similar to the excitatory neurotransmitter glutamate (Schwarz, 2016) and binds to glutamatergic receptors with a K_i_ of 2.1 μM *in vitro* (Olverman et al., 1984). Recently, SSC was shown to stimulate glutamatergic receptors in cell culture (Kumar et al., 2017). SSC is highly abundant in MoCD and isolated SOX deficiency patients (Schwarz, 2016). Here, it causes neuronal hyperexcitation resulting in severe seizures in newborns, neuronal cell death, and finally lethal brain damage (Johnson and Duran, 2001; Schwahn et al., 2015). Interestingly, MoCD and isolated SOX deficiency can be diagnosed immediately after birth by a urine test for SSC (Belaidi et al., 2012). Hence, there is a therapeutic window for reverting disease conditions. Until today, only one MoCD therapy method exists (Schwahn et al., 2015; Schwarz, 2016), which is exclusively suitable for patients harboring defects in the first Moco biosynthesis pathway step (MoCD Type A). However, no therapy is available for patients suffering under either MoCD caused by mutations affecting the second or third step of the Moco biosynthesis pathway (MoCD Types B and C) or isolated SOX deficiency (Johnson and Duran, 2001). Development of a suitable therapy for the general lack of SOX activity is hence of the highest importance. Murine model systems for MoCD Types A (Lee et al., 2002) and B (Jakubiczka-Smorag et al., 2016) are available; however, the low number of progeny and also ethical concerns preclude their use in systematic drug screening addressing the SSC-induced phenotype in MoCD or isolated SOX deficiency patients. Severe seizures are the most prominent phenotype of MoCD (Schwahn et al., 2015). Zebrafish larvae possess the brain structures necessary to display seizure-like behavior (Baraban et al., 2005). Therefore, it seems reasonable to test zebrafish as a model for MoCD and isolated SOX deficiency. Zebrafish produces up to 300 eggs per week; the embryos and larvae display a rapid *ex utero* development and are generally permeable to small water-soluble compounds, making zebrafish accessible for high-throughput screening. In this study, we present a SSC-induced phenotypic model for MoCD and isolated SOX deficiency in zebrafish.

## Materials and Methods

### Zebrafish Strains and Husbandry

Zebrafish wild-type strain AB was used for all experiments. All animals were maintained according to standard procedures (Westerfield, 1995) and in accordance with legal regulations (EU-Directive 2010/63), the local authorities, the animal welfare representative of the Braunschweig University of Technology, and the Lower Saxony State Office of Consumer Protection and Food Safety (LAVES, Oldenburg, Germany; Az. §4 (02.05) TSchB TU BS).

### Compounds

SSC (Sigma Aldrich) was dissolved at a concentration of 50 mM in distilled water. MK801 (Sigma Aldrich) was dissolved at a concentration of 10 mM in distilled water.

### Toxicity Tests

To determine the median lethal concentration of SSC, 20 one- or two-cell embryos were transferred into fresh 30% Danieau medium (290 mM NaCl, 7 mM KCl, 4 mM MgSO_4_, 6 mM Ca(NO_3_)_2_, 50 mM HEPES buffer, pH = 6.8) and used directly or raised until 24, 55, or 72 hours post fertilization (hpf). The medium was changed daily. For SSC treatment, the medium was removed and replaced by different concentrations of SSC in 30% Danieau reaching from 0 to 4 mM. Embryos and larvae were analyzed for 24 h.

### Acridine Orange (AO) Staining

Upon SSC treatment, 3 days post fertilization (dpf) larvae were washed 3× with 30% Danieau. Staining was carried out with 10 μg/mL AO (Sigma Aldrich) in 30% Danieau for 1 h in the dark at room temperature. Prior to imaging, larvae were washed 3× with 30% Danieau. Larvae were anesthetized with 0.04% tricaine mesylate (Sigma Aldrich) in 30% Danieau and embedded in 1.5% low melting agarose dissolved in 30% Danieau.

### Behavioral Experiments

About 3 dpf larvae were exposed to SSC. Mobility of all larvae used for tracking experiments was previously verified. For each experiment, five larvae per well were transferred into fresh 30% Danieau and allowed to habituate 10 min prior to SSC application. SSC was added to a final concentration of 2 mM, and the larvae were allowed to accommodate for 30 s. SSC was either applied alone or in combination with 2 mM MK801. For the control, an equivolume of 30% Danieau was applied. To analyze the effect of SSC, the motor behavior was monitored using the Samsung Galaxy A3 Smartphone camera with a resolution of 13 megapixel at 30 frames per s adapted to a stereo microscope (LEICA S8APO) utilizing the DKA5 Smartphone adapter (recording time: 9 min). Tracking experiments were performed in self-made agarose well plates (1.5% agarose in 30% Danieau heated and solidified and stamped out wells of 16 mm in diameter). To track the movement, movies were analyzed framewise using the Manual Tracking plugin of ImageJ (Schindelin et al., 2012). Measuring points were placed between the eye balls of the analyzed larva. X/Y coordinates were used to analyze and quantify the motor behavior. The traveled distance between frames was calculated by using the difference in X/Y positions between the frames, and the percentage of time spent with movement was determined with calculating the amount of frames, where the distance traveled was >0. Statistical analysis was carried out using a one-tailed unpaired *t* test.

### Inactivation of Sensory Hair Cells

Inactivation of sensory hair cells of the lateral line (Harris et al., 2003) was carried out by treatment with 400 μM neomycin in 30% Danieau for 1 h and subsequent washing 3× in 30% Danieau. Ablated cells were confirmed by staining with 0.05% DASPEI in 30% Danieau for 15 min.

## Results

### SSC Induces Seizure-Like Behavior and Neurodegeneration in Zebrafish

**Figure 1:**
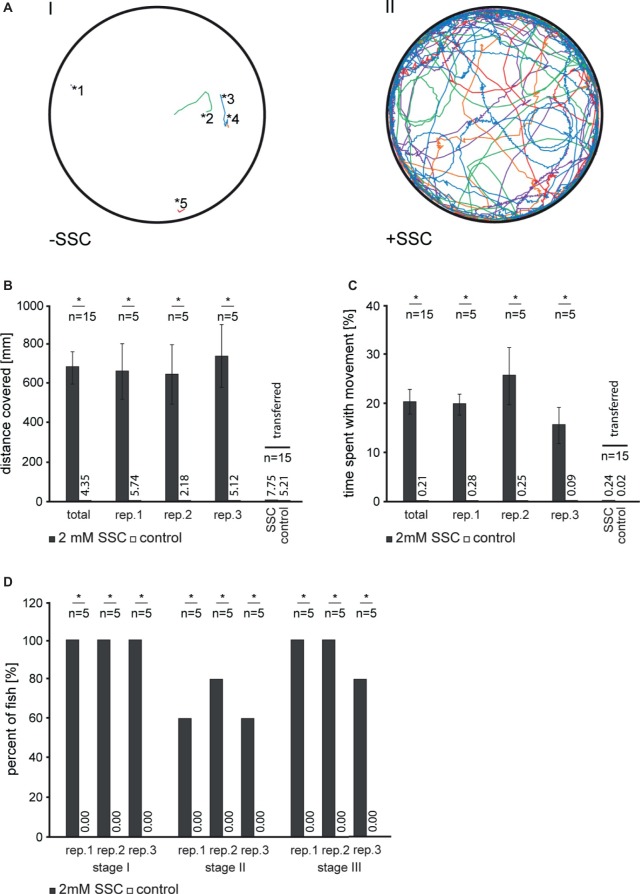
Analysis of S-Sulfocysteine induced movement in 3 dpf zebrafish larvae. **(A)** For each experiment, routinely five larvae per well were transferred in fresh 30% Danieau and allowed to habituate 10 min prior to S-Sulfocysteine (SSC) application. SSC was added to a final concentration of 2 mM (right, II). As a control (left, I), an equivolume of 30% Danieau was added. Larvae were allowed to accommodate for 30 s and then imaged for 9 min. The recorded tracks for each of the larvae analyzed are shown in different colors. The starting points of non-treated larvae are illustrated by asterisks with the corresponding numbers of the larvae. **(B–D)** Seizure-like behavior of SSC-treated larvae was quantified as described (Baraban et al., 2005). **(B)** For stage I, the total distances traveled for all larvae were determined and plotted. **(C)** Percentage time larvae spent with movement over the time span of the experiment. In an additional experiment, larvae were transferred to fresh medium after 10 min incubation with SSC. All data obtained for the transfer experiment were summarized in one bar chart. Error bars indicate the standard error of the mean. **(D)** Percentage of larvae showing increased activity (stage I), circling swim behavior (stage II), and clonus-like convulsions (stage III). For all experiments, three full independent biological replicates (rep.) were analyzed; *, significant with *p* < 0.01.

To test for the effect of SSC on zebrafish motor behavior, we applied 2 mM SSC to 3 dpf larvae and monitored the acute effects. SSC-treated larvae showed an increase in general swimming activity accompanied by circling and convulsive episodes (Figure 1A). We quantified these seizure-like behaviors (Baraban et al., 2005): SSC-treated larvae traveled a significantly longer distance and spent more time with movement as non-treated larvae (Figures 1B,C), hence conforming to “stage I: dramatically increased swim activity.” Also “stage II: rapid ‘whirlpool-like’ circling” and “stage III: brief clonus-like convulsions” were drastically enhanced (Figure 1D). These effects were reversible as demonstrated by the normalized swimming behavior of SSC-treated larvae, which were transferred back to SSC-free medium (Figures 1B,C). To exclude effects of SSC on the peripheral nervous system *via* the sensory hair cells of the lateral line, we ablated these cells; successful hair cell ablation was confirmed by DASPEI staining [Supplementary Figure S1; (Harris et al., 2003)]. These larvae likewise showed a seizure-like phenotype when 2 mM SSC was applied (Supplementary Video S1) with the corresponding control showed no increased movements (Supplementary Video S2). These findings indicate that the observed seizure-like behaviors are likely caused by an SSC-induced stimulation of the central nervous system. To characterize the observed movements in detail, representative continuous frames were analyzed (Supplementary Figures S2,S3) with an extract of the image sequence of SSC-treated and non-treated larvae being shown in Supplementary Figure S4. Notably are episodes of involuntary erratic movements with aberrant bending of the body axis differentiating the SSC-induced phenotype from the sine-like curved axis during movement of control larvae. Prolonged exposure of larvae to SSC is lethal with the SSC LC_50_ values varying depending on developmental stages (Supplementary Figure S5). Consistently, increasing levels of cell death in the central nervous system were observable in larvae upon prolonged incubation with SSC (Supplementary Figure S6A). Here, the heads of SSC-treated zebrafish larvae were also found to engorge (Supplementary Figures S6A,B), which might correspond to cerebral edema and increased brain pressure in MoCD patients (Durmaz and Özbakir, 2018).

### An *N*-Methyl-d-Aspartate (NMDA) Receptor Antagonist Partially Compensates SSC-Triggered Movements in Zebrafish Larvae

**Figure 2:**
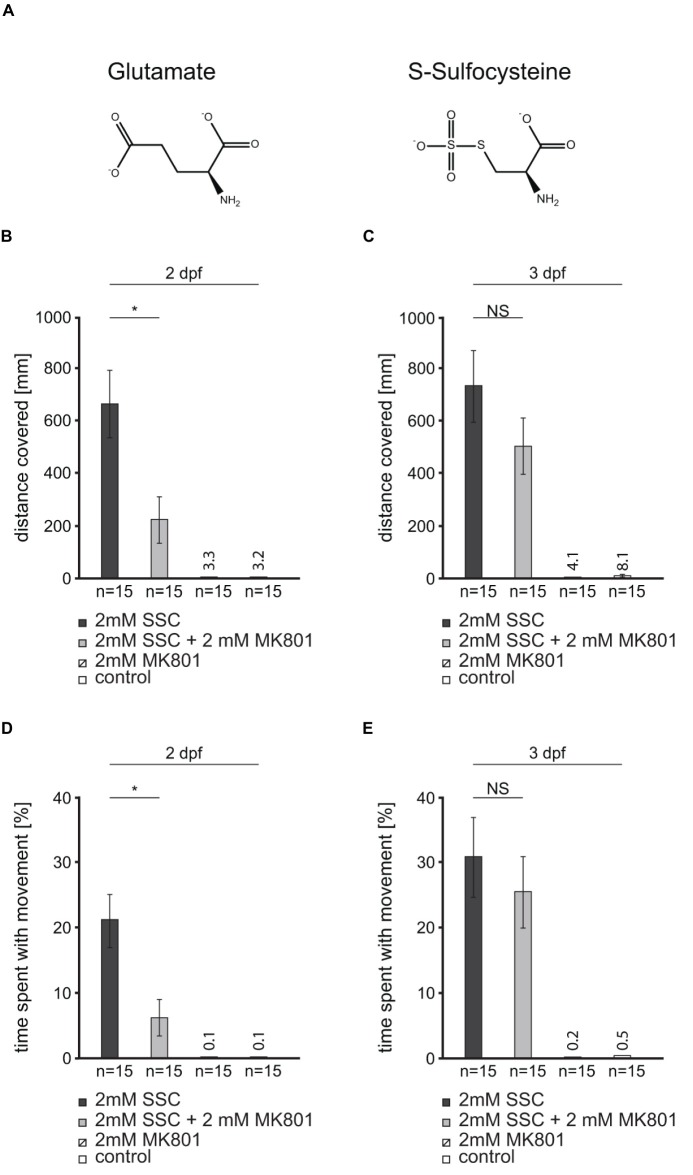
An *N*-methyl-d-aspartate (NMDA) receptor antagonist partially rescues the S-Sulfocysteine induced seizure-like behavior in zebrafish. **(A)** Glutamate and S-Sulfocysteine (SSC) are structurally highly similar (Schwarz, 2016). **(B–E)** The effect of the NMDA receptor antagonist MK801 on SSC-induced zebrafish movement was characterized by the addition of 2 mM MK801 to 2 and 3 dpf larvae treated with 2 mM SSC. **(B)** and **(C)** The total distance traveled by SSC-treated 2 dpf and 3 dpf larvae was determined and compared to the distance traveled in the presence of SSC + MK801. As controls, larvae were treated with MK801 and water, respectively. **(D)** and **(E)** Percentage of time SSC-treated and SSC + MK801-treated larvae spent with movement. The controls were the same as for **(B)** and **(C)**. Three full independent biological replicates were analyzed. Error bars represent the standard error of the mean. NS, not significant; *, significant with *p* < 0.01.

The NMDA receptor antagonist MK801 was found to partially compensate for excitotoxicity caused by the glutamate analog SSC (Figure 2A) in mammalian cell culture and mice (Kumar et al., 2017). To test for NMDA receptor antagonist efficacy in SSC-treated zebrafish, we applied the non-competitive NMDA receptor antagonist MK801 to 2 and 3 dpf larvae. Upon application to 2 dpf larvae, the afore described seizure-like behavior (Figure 1) was largely compensated as documented by normalized larval movement (Figures 2B,D), reduced clonus-like convulsions, and diminished circling behavior. Therefore, MK801 can partially compensate the SSC-triggered movements. When 3 dpf larvae were co-treated, the compensation of the SSC-induced phenotype was less pronounced and not significant (*p* = 0.1004, Figure 2C; *p* = 0.256, Figure 2E), probably because of diminished MK801 penetrance into the brain with ongoing larval differentiation. Nevertheless, these findings in conjunction with the well-known binding and the activation of mammalian NMDA receptors by SSC (Olverman et al., 1984; Kumar et al., 2017) suggest that SSC-mediated hyperactivation of NMDA receptors is conserved in zebrafish.

## Discussion

In this study, we describe the effect of SSC on zebrafish larvae. SSC-treated larvae were found to display all qualitative and quantitative characteristics defined for seizure-like behavior (Baraban et al., 2005) as demonstrated by visual and statistical evaluations, hence leading us to the conclusion that SSC can be used to chemically induce a highly reproducible and therefore robust phenotypic model for MoCD and isolated SOX deficiency in zebrafish. Prior to this, the effects of excessive SSC in the central nervous system and other tissues of vertebrates were studied directly in the patients suffering from MoCD or isolated SOX deficiency or in mice carrying *loss-of-function* mutations in the respective genes (Johnson and Duran, 2001; Lee et al., 2002; Belaidi et al., 2012; Jakubiczka-Smorag et al., 2016; Schwarz, 2016; Durmaz and Özbakir, 2018). As reported for murine cell culture (Kumar et al., 2017), high SSC concentrations have a toxic effect on zebrafish larvae. Moreover, SSC LC_50_ values were found to vary, depending on the developmental stage, suggesting a development-related sensitivity. Interestingly, prolonged exposure to SSC results in massive cell death in the central nervous system and also possibly in cerebral edema, which is well known for patients suffering from MoCD (Durmaz and Özbakir, 2018). Therefore, the phenotypic effects of MoCD and isolated SOX deficiency can now be studied in a concentration and development-related manner. The effects of SSC on zebrafish larvae can be partially rescued by the application of the non-competitive NMDA receptor antagonist MK801. The compensatory potential of MK801 is at most in 2 dpf larvae, and its reduced compensatory potential for 3 dpf larvae may be best explained by lowered permeability at that developmental stage. Also, blood-brain barrier differentiation or a diverging subunit composition of zebrafish NMDA receptors at different developmental stages may be causal (Fleming et al., 2013; Meneszes et al., 2015). A compensatory effect of MK801 was already described for SSC-treated mouse neurons in culture (Kumar et al., 2017). These findings suggest that in zebrafish, the underlying principles of SSC-triggered excitotoxicity may be related to the respective processes in mammals. Furthermore, these results show that zebrafish larvae may be used to screen for compounds to rescue or alleviate the SSC-induced effect with anti-seizure or anti-epileptic drugs being most obvious. Such screenings have been already successfully performed with a chemical-induced zebrafish model with a focus on the inhibitory neurotransmitter gamma-aminobutyric acid (Baraban et al., 2005; Afrikanova et al., 2013). Using zebrafish as a model system to study the effects of SSC neurotoxicity paves the way for the systematic characterization of SSC-related effects on the developing vertebrate nervous system. The value of such chemical-induced disease models for not only fundamental research but also drug development is long known with Morbus Parkinson being a prominent example (Hisahara and Shimohama, 2011). Not only various already developed therapeutics but also pre-clinical substances may be easily and effectively tested with respect to their potential to compensate SSC-induced phenotypes in zebrafish.

## Author Contributions

JP, WS, RK, and TK drafted the article. JP, WS, and TK contributed to the conception and design. JP and FL participated in the acquisition of data. JP, WS, AH, and TK analyzed and interpreted the data. RK and TK approved the final version of the manuscript.

### Conflict of Interest Statement

The authors declare that the research was conducted in the absence of any commercial or financial relationships that could be construed as a potential conflict of interest.

## Supplementary Material

SUPPLEMENTARY VIDEO S1 Motor behavior of S-Sulfocysteine treated zebrafish larvae with previously ablated hair cells. In 3 dpf zebrafish larvae, the sensory hair cells of the lateral line were ablated by treatment with 400 μM neomycin in 30 % Danieau medium for 60 min. After confirmation of the ablation by staining with 0.05 % DASPEI in 30 % Danieau for 15 min (Supplementary Figure S1), 5 larvae per well were transferred in fresh 30 % Danieau and allowed to habituate 10 min prior to S-Sulfocysteine (SSC) application. SSC was added to a final concentration of 2 mM. Three independent biological replicates were analyzed with a representative video of the observed phenotype being shown.

SUPPLEMENTARY VIDEO S2 Motor behavior of zebrafish larvae with previously ablated hair cells. In 3 dpf zebrafish larvae, the sensory hair cells of the lateral line were ablated by treatment with 400 μM neomycin in 30 % Danieau medium for 60 min. After confirmation of the ablation by staining with 0.05 % DASPEI in 30 % Danieau for 15 min (Supplementary Figure S1), 5 larvae per well were transferred in fresh 30 % Danieau and allowed to habituate 10 min. As these larvae served as a control for the S-Sulfocysteine treatment carried out (Supplementary Video S1), an additional equivolume of 30 % Danieau was applied. Three independent biological replicates were analyzed with a representative video of the observed phenotype being shown.
