# Supplementary figures and images for: S-Sulfocysteine Induces Seizure-Like Behaviors in Zebrafish
Source: Front Pharmacol. 2019 Apr 2;10:122. doi: 10.3389/fphar.2019.00122 (PMC6454129; doi:10.3389/fphar.2019.00122)

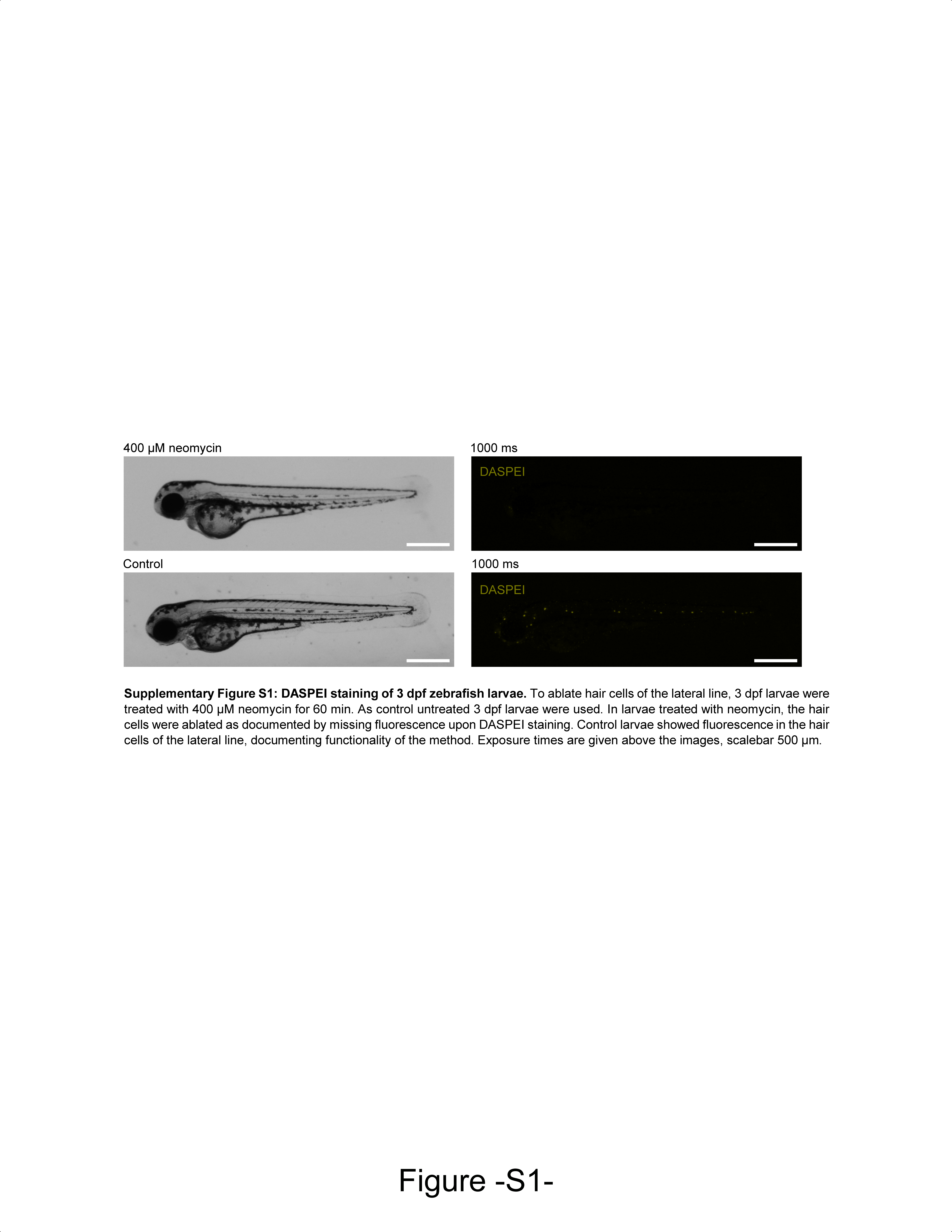

Supplement: Supplementary file 1 [file Image_1.tif]

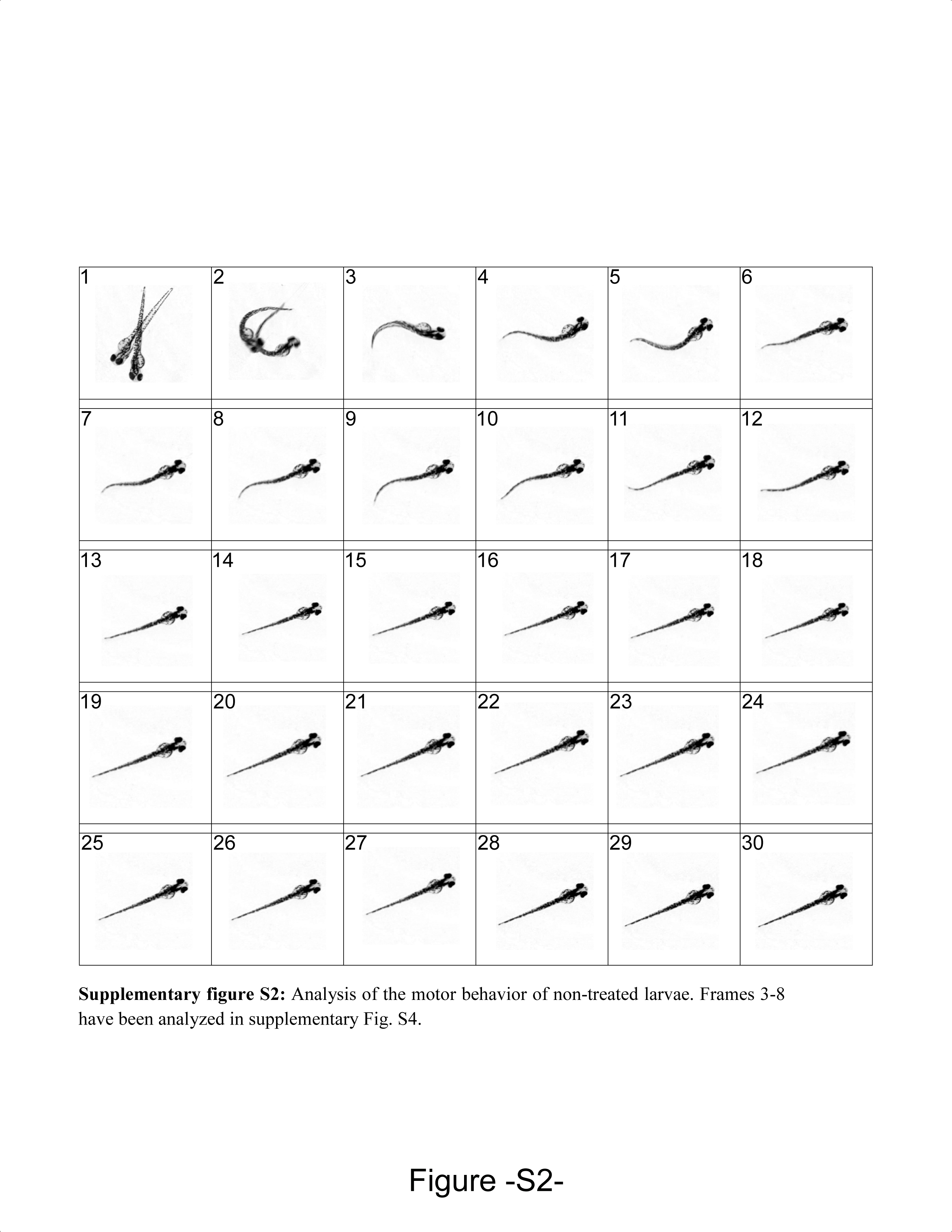

Supplement: Supplementary file 2 [file Image_2.TIF]

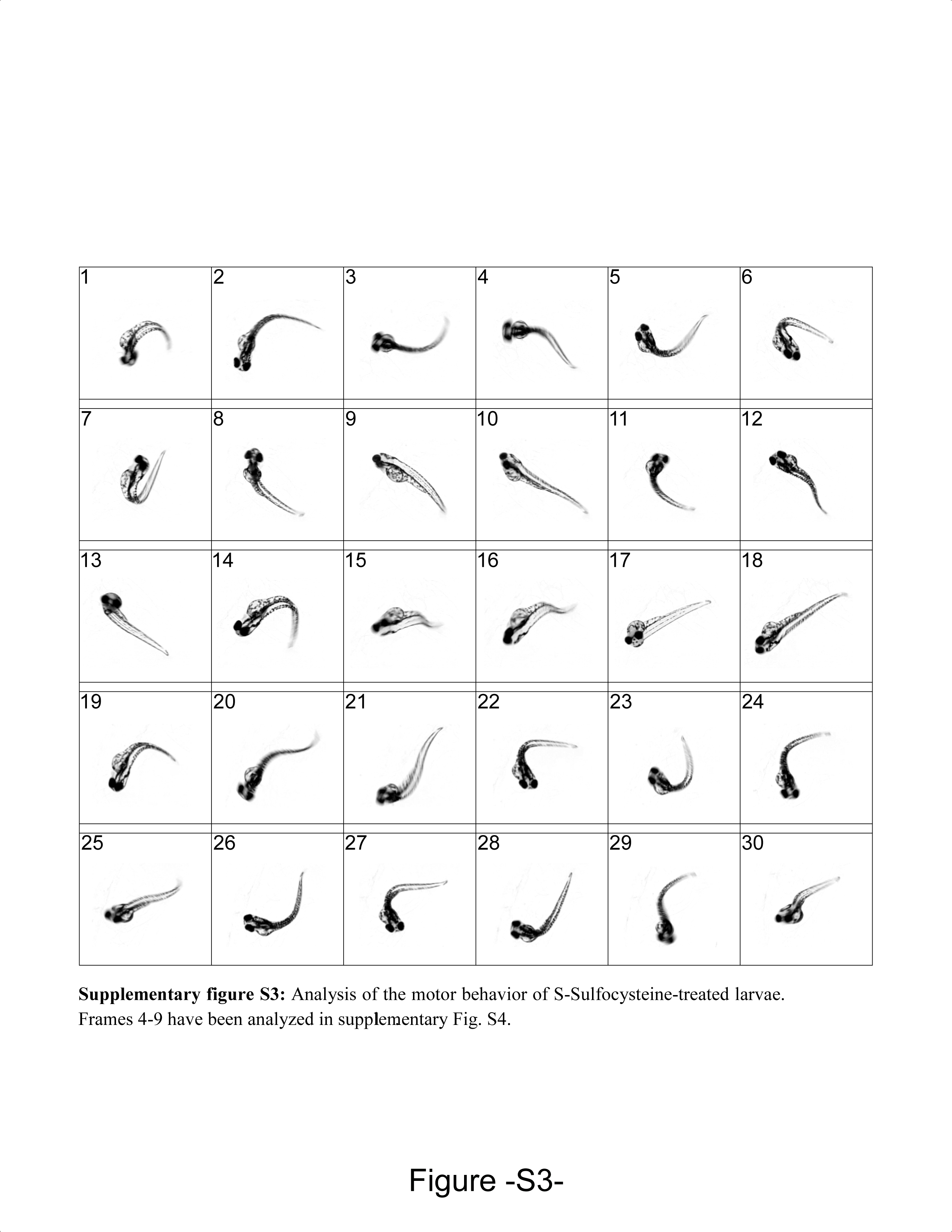

Supplement: Supplementary file 3 [file Image_3.TIF]

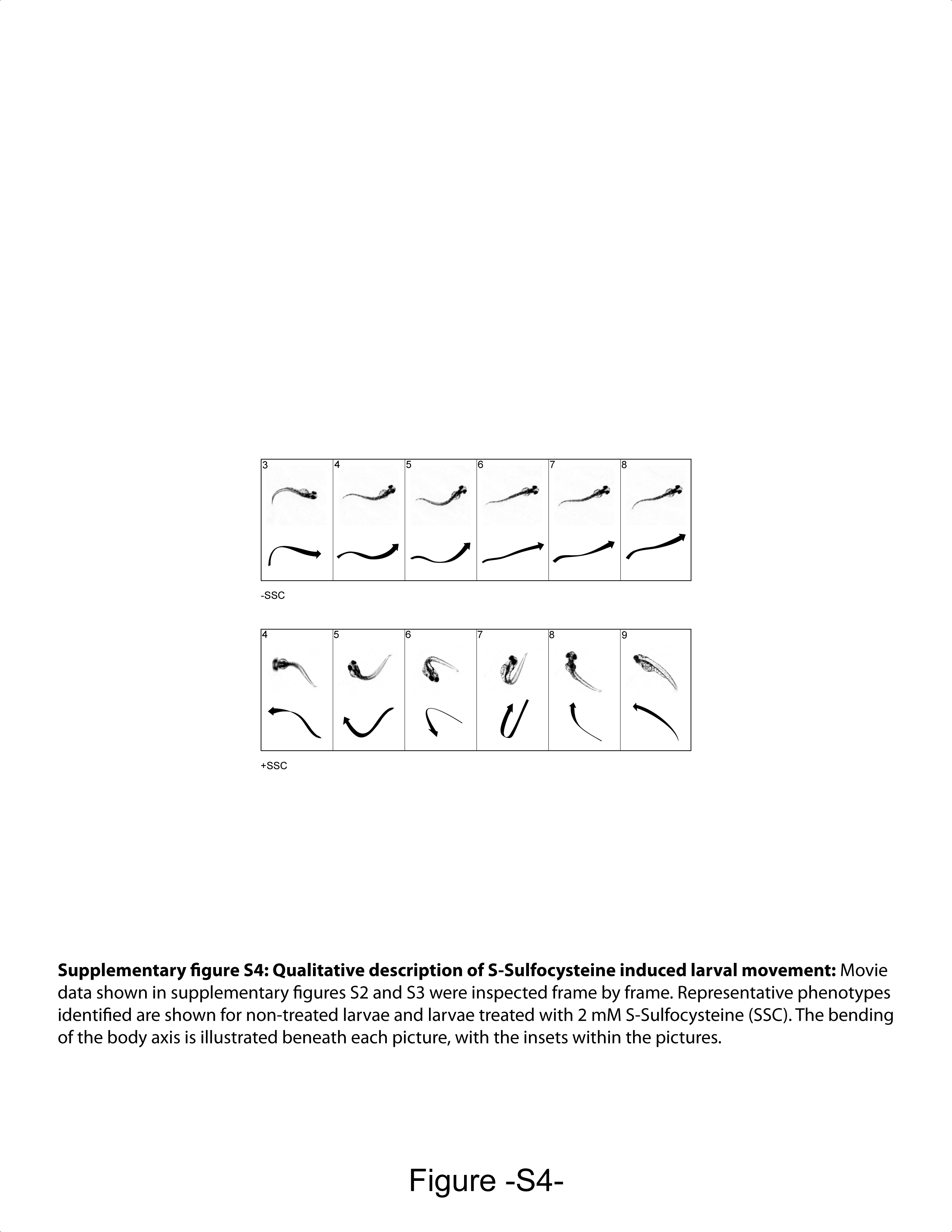

Supplement: Supplementary file 4 [file Image_4.TIF]

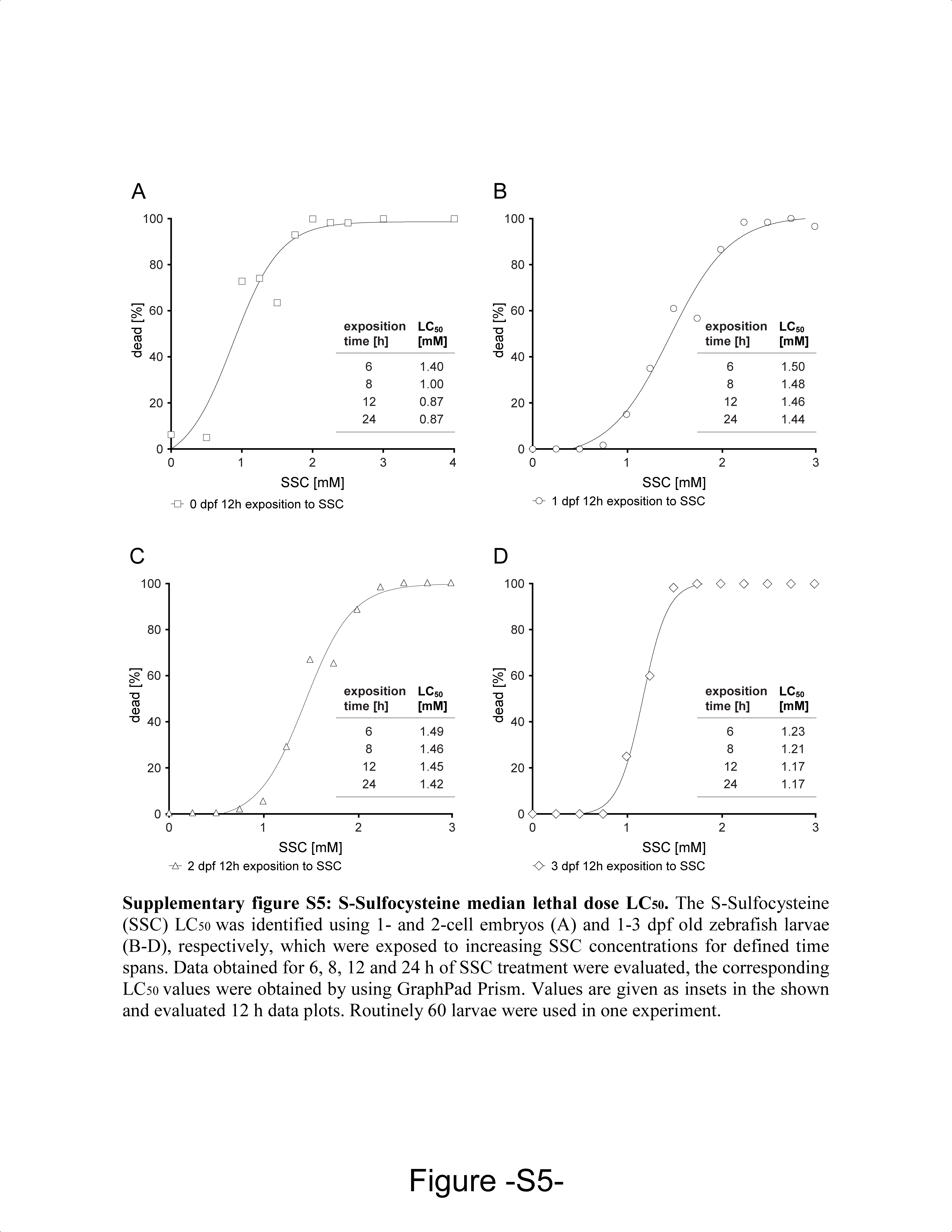

Supplement: Supplementary file 5 [file Image_5.tif]

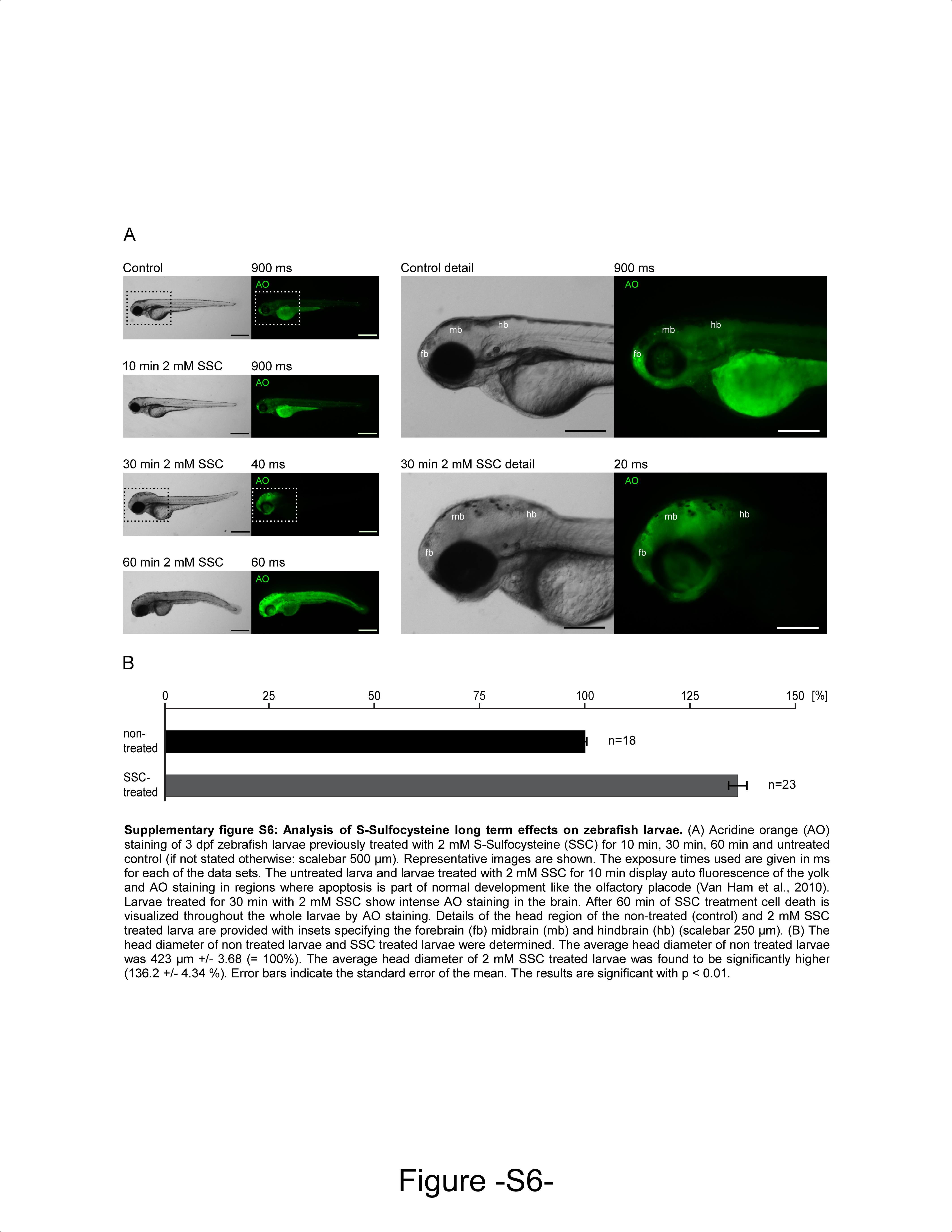

Supplement: Supplementary file 6 [file Image_6.tif]
